# Nipah Virus Transmission from Bats to Humans Associated with Drinking Traditional Liquor Made from Date Palm Sap, Bangladesh, 2011–2014

**DOI:** 10.3201/eid2204.151747

**Published:** 2016-04

**Authors:** M. Saiful Islam, Hossain M.S. Sazzad, Syed Moinuddin Satter, Sharmin Sultana, M. Jahangir Hossain, Murshid Hasan, Mahmudur Rahman, Shelley Campbell, Deborah L. Cannon, Ute Ströher, Peter Daszak, Stephen P. Luby, Emily S. Gurley

**Affiliations:** Icddr,b, Dhaka, Bangladesh (M.S. Islam, H.M.S. Sazzad, S.M. Satter, M.J. Hossain, M. Hasan, S.P. Luby, E.S. Gurley);; Institute of Epidemiology Disease Control and Research, Dhaka (S. Sultana, M. Rahman);; Medical Research Council, London, United Kingdom (M.J. Hossain);; Centers for Disease Control and Prevention, Atlanta, Georgia, USA (S. Campbell, D.L. Cannon, U Ströher);; EcoHealth Alliance, New York, New York, USA (P. Daszak);; Stanford University Center for Innovation in Global Health, Stanford, California, USA (S.P. Luby)

**Keywords:** Nipah virus, NiV, fermented date palm sap, tari, Pteropus, bats, Bangladesh, paramyxovirus, viruses

## Abstract

Nipah virus (NiV) is a paramyxovirus, and *Pteropus* spp. bats are the natural reservoir. From December 2010 through March 2014, hospital-based encephalitis surveillance in Bangladesh identified 18 clusters of NiV infection. The source of infection for case-patients in 3 clusters in 2 districts was unknown. A team of epidemiologists and anthropologists investigated these 3 clusters comprising 14 case-patients, 8 of whom died. Among the 14 case-patients, 8 drank fermented date palm sap (*tari*) regularly before their illness, and 6 provided care to a person infected with NiV. The process of preparing date palm trees for *tari* production was similar to the process of collecting date palm sap for fresh consumption. Bat excreta was reportedly found inside pots used to make *tari*. These findings suggest that drinking *tari* is a potential pathway of NiV transmission. Interventions that prevent bat access to date palm sap might prevent *tari*-associated NiV infection.

## Abstract 2

Interventions that prevent bat access to this sap might prevent these infections.

Nipah virus (NiV) is a bat-borne emerging infection, and *Pteropus* spp. bats are the wildlife reservoir (*1*). NiV was discovered in an outbreak in Malaysia in 1998 that affected 283 persons and caused 109 deaths (case-fatality rate 39%) (*2*). Subsequently, outbreaks of NiV infection have occurred nearly every year in Bangladesh and occasionally in India (*1*,*3*–*5*). A total of 33 outbreaks of NiV encephalitis were reported in Bangladesh and India during 2001–2014, and epidemiologic investigations implicated batborne and human-to-human transmission (*6*,*7*). During 2004–2012, a total of 157 NiV infections were reported in Bangladesh, and 22% of these occurred through human-to-human transmission (*8*).

Investigations of NiV-associated outbreaks in Bangladesh identified consumption of fresh date palm sap as the primary route of bat-to-human transmission (*1*,*9*). In Bengali culture, sap harvested from the date palm tree is commonly used for fresh consumption and fermentation (*10*,*11*). Moreover, in Asia, Australia, and Africa, fermented date palm sap is used to make alcoholic drinks, known as toddy, *tari*, or palm wine (*12*,*13*). In Bangladesh, date palm sap is typically collected in clay pots that are attached to the tree. A top section of the date palm tree bark is shaved, allowing the sap to ooze overnight into the collection pot (*11*). A previous NiV study reported that *Pteropus* spp. bats frequently feed on the shaved bark and often contaminate the sap with saliva, urine, and excreta (*14*). *Pteropus* spp. bats are also known to occasionally shed NiV in their secretions and excretions (*15*,*16*).

Since 2006, the Institute of Epidemiology, Disease Control, and Research (IEDCR) in Dhaka, Bangladesh, under the Ministry of Health and Family Welfare of Bangladesh, has collaborated with the icddr,b, Dhaka, on hospital-based encephalitis surveillance in the areas where NiV-associated outbreaks have been reported (*3*). From December 2010 through March 2014, the surveillance identified 18 clusters of NiV infection; in 15 of these clusters, the index case-patients had exposure to fresh date palm sap before illness onset. For the remaining 3 clusters, the index case-patients had no known contact with date palm saps, bats, or sick animals other than bats. Recognizing the potential for new pathways of transmission, we investigated other possible exposures to NiV by applying epidemiologic and anthropological approaches in our study of these 3 NiV disease clusters. We used the epidemiologic study to explain the proximate individual-level factors linked to the disease outbreak (*17*) and an anthropological approach to explicate local perceptions, behaviors, and practices that might have contributed to the disease occurrence (*18*). Therefore, the objectives of our investigation were to describe the clinical signs and symptoms of the case-patients and determine the possible route of transmission for these clusters.

## Methods

The team conducted this study during 2011–2014 in Rajshahi and Rangpur Districts, Bangladesh. Surveillance physicians from Rangpur Medical College Hospital and Rajshahi Medical College Hospital identified suspected case-patients (Table), recorded clinical histories and home addresses, and collected whole blood samples from each. The serum was separated from each sample, stored in liquid nitrogen at the hospital, and then transported to IEDCR (*3*). The laboratory team at IEDCR tested serum samples for NiV IgM and IgG by using IgM-capture and indirect IgG enzyme immunoassays (*19*,*21*).

**Table:** Case definitions for Nipah virus (NiV) infections that occurred in 3 clusters, Rangpur and Rajshahi Districts, Bangladesh, 2011, 2012, and 2014

Type of case	Case definition
Suspected	Fever or history of fever with axillary temperature >38.5°C, altered mental status, new onset of seizures, or a new neurologic deficit in a patient from an adult or pediatric ward of an NiV surveillance hospital during the NiV season (*3*).
Probable	Illness meeting the case definition for suspected NiV infection in a person who lived in the same village as a person with laboratory-confirmed NiV infection but who died before specimens could be collected for diagnosis (*19*).
Laboratory-confirmed	Acute onset of fever and subsequent altered mental status or other neurologic deficits during the outbreak period and having NiV IgM or IgG antibodies in serum (*19*).
Primary	A case in which illness occurred in the absence of contact with a symptomatic case-patient.
Secondary	Illness in a person whose only known exposure was to a case-patient and whose illness occurred within 5–15 d after that contact (*20*).


On the basis of type of exposure, we categorized suspected case-patients as primary or secondary case-patients, and these two groups were further categorized as case-patients with probable or laboratory-confirmed NiV infection (Table). To identify a potential NiV infection cluster, surveillance physicians asked each of the admitted suspected case-patients and their caregivers present in the hospital about other sick persons or persons in their communities who had recently died with similar symptoms. We defined a NiV infection cluster as >2 suspected meningoencephalitis case-patients living within 30 minutes’ walking distance from each other who had onset of similar illnesses within 3 weeks of one another or had epidemiologic linkages to one another (*22*).

After identifying laboratory-confirmed NiV infection cases (Table), the team reviewed hospital records, took preliminary information from the surveillance physicians, and visited case-patients from each cluster within a month of case confirmation. We limited this study to clusters in which no case-patients had a history of drinking fresh date palm sap. In the community, the team used a structured questionnaire to interview surviving case-patients and the friends, relatives, and neighbors of deceased case-patients as proxy respondents. The team collected information related to exposure and signs and symptoms of illness to determine routes of NiV transmission for each case-patient.

The team visited the households of case-patients and used culturally appropriate approaches to build rapport and trust with the community (*23*). The team conducted in-depth interviews and group discussions with surviving case-patients and the family caregivers, friends, and neighbors of the deceased to explore the exposure histories of each case-patient by using an open-ended interview guide. Open-ended questions allowed the interviewers to obtain new insights about the outbreak. Good rapport with community members helped the team collect information on potentially sensitive issues, such as alcohol consumption (*24*), which is prohibited among the majority Muslim population of Bangladesh. The preliminary data collected during this study suggested that the case-patients might have consumed *tari* before their illness onset. Therefore, the team also interviewed 5 date palm sap harvesters and conducted 3 group discussions with community members to learn about *tari* production, consumption, and selling practices in the affected communities. The team also collected and analyzed whole blood samples from surviving case-patients and from nonpatients (defined as persons in the community who drank *tari* with the case-patients but did not experience any symptoms) by using the same methods described above.

### Data Analysis

We used descriptive statistics to characterize the demographic and clinical characteristics of the case-patients. The team expanded the observation and interview field notes and summarized them. The primary author (M.S.I.) read summaries of the interviews and identified themes. These themes were shared among investigators for review and consensus. The primary author then categorized the data according to the selected themes, consistent with methods previously described (*25*).

### Ethical Considerations

The team obtained verbal informed consent from study participants. The surveillance and outbreak investigation study protocol was reviewed and approved by the ICDDR,B Ethical Review Committee.

## Results

Three clusters were identified, consisting of 14 case-patients (9 with confirmed NiV infection, 5 with probable NiV infection). Eight of the 14 case-patients were primary case-patients (3 with confirmed NiV infection, 5 with probable NiV infection). All 6 of the secondary case-patients had confirmed NiV infection. Among the 14 case-patients, 7 had illness onset during January–March 2011 in Rangpur District (first cluster), 3 had illness onset in February 2012 in Rajshahi District (second cluster), and 4 had illness onset during January–February 2014 in Rangpur District (third cluster). Eight drank *tari* before their illness onset, whereas 6 only had exposure to other case-patients. None of the primary case-patients had any history of drinking fresh date palm sap or exposure to sick humans or animals. All of the illnesses began with fever. All 8 of the primary case-patients had altered mental status followed by loss of consciousness and death. The median duration from illness onset to death was 6 days. All 6 secondary case-patients survived. The median age of all 14 case-patients was 32 years. All the primary case-patients were male, and all the secondary case-patients were female. None of the non–case-patients had NiV IgM or IgG detected in their serum samples.

In the 2011 Rangpur cluster, case-patients A, B, C, and G drank *tari* regularly in the evenings, and the sap to make *tari* was collected from a single village (Figure). Case-patients A and G were family friends and lived in the same village. Case-patients B and C were also related and lived in the same village. Case-patients D, E, and F were family caregivers of case-patient C and provided close-contact care during his illness. All 3 family caregivers experienced illness within 2 weeks of case-patient C’s illness onset.

**Figure:**
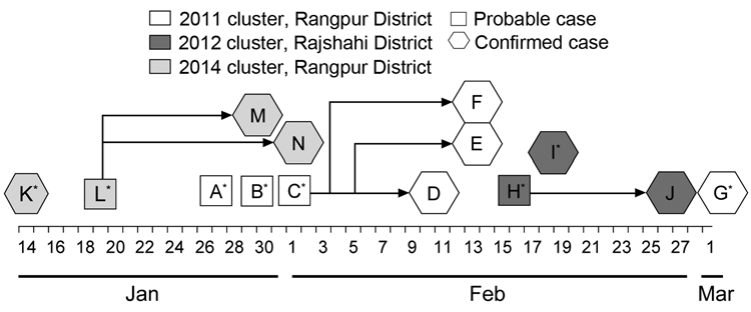
Timeline of illness onset in persons with primary and secondary cases of Nipah virus infection that occurred in 3 clusters, Rangpur and Rajshahi Districts, Bangladesh, 2011, 2012, and 2014. Asterisks indicate primary cases; cases without an asterisk are secondary cases.

In the 2012 Rajshahi cluster, case-patient H was a *tari* producer and harvester of date palm sap. Case-patient I was a neighbor and cousin of case-patient H, and both drank *tari* regularly in the evenings. They drank *tari* together from the same pot 6 days before their illness onsets. Case-patient J was a family caregiver of patient H and provided close-contact care during his illness. She experienced fever 11 days after case-patient H’s illness onset.

In the 2014 Rangpur cluster, the respondents reported that case-patients K and L were family friends and had drunk *tari* every day in the evenings before their illness onsets (Figure). Case-patient L became ill after 5 days after case-patient K, and case-patient L did not have any contact with case-patient K while case-patient K was ill. The *tari* they drank was collected from the same village where the 2011 Rangpur cluster was identified. Case-patients M and N shared the same bed with case-patient L at home; case-patient N was a family caregiver to case-patient L, providing close-contact care for him at home and in the hospital. Case-patient M had onset of fever 12 days later, and case-patient N had onset of fever 14 days after case-patient L’s illness onset.

### *Tari* Production, Processing, and Selling Patterns in Rangpur and Rajshahi

The area of Rangpur where the case-patients lived was well known for its *tari* production and was the largest *tari*-producing area in the district. An estimated 500 date palm trees grow in the area, which produces and supplies *tari* throughout the district. *Tari* retailers from different subdistricts of Rangpur also come to this area to buy *tari* at a wholesale price. In the affected villages, the *tari* producers reported that they had been leasing all the date palm trees for several years. Villagers reported they did not have regular access to fresh date palm sap because almost all the date palm sap from the village was made into *tari*.

In the NiV-affected area of Rajshahi, the *tari* is produced in small quantities. An estimated 15 date palm trees grow in the affected community of Rajshahi. The *tari* producer leases these trees only during winter to produce *tari*.

The *tari* production process was identical in each of the affected communities. The process of preparing the date palm trees for *tari* production was similar to the process for collecting date palm sap for fresh consumption (*11*). To prepare the tree for tapping, the sap harvesters cut the old leaves close to the top of the trees with a knife to expose the tender part of the tree. To tap the tree, the harvesters shave a V-shaped cut at the top of the tree and set a bamboo spigot at the end of the cut. After 3–5 days, the V cut is shaved again, and an earthen pot is hung under the spigot to catch the sap that oozes out. For fresh date palm sap, the harvesters need to clean and dry the sap collection pots after each episode of sap collection. However, *tari* harvesters from both affected communities reported that they use the same earthen pot for sap collection for several days without cleaning it so that yeast can form at the bottom of the earthen pot. Yeast aids the fermentation of the fresh date palm sap in the pot.

In Rangpur, the date palm sap is harvested for *tari* year round. The harvesters reported that date palm sap can be collected from each tree for 3–4 days a week for 4 months at a time, and then the tree is left to recover for the next 7–8 months. The harvesters stagger the tapping of trees so there is a continuous supply of date palm sap to make *tari* throughout the year. The harvesters reported that they harvest more sap during winter than during other seasons because of higher demand for *tari* from consumers. Moreover, they reported that the sap flows more freely from each tree during the winter. In the affected communities, the harvesters reported that every day from 8 a.m. until noon, they collect the sap from the hanging earthen pots and accumulate the collected sap in other earthen pots or containers and leave the hanging pots on the trees. In Rangpur, the harvesters bring *tari* to their house and immediately sell it to retailers and consumers from morning until late at night. Occasionally, consumers take *tari* away with them in plastic bottles. In the affected area of Rajshahi, after being removed from the trees, the *tari* pots are kept in a betel leaf garden, and the harvesters sell *tari* from there.

The *tari* sellers and harvesters reported that *tari* consumers are men and included truck and bus drivers, day laborers, rickshaw pullers, and local farmers. They also reported that *tari* is less expensive than other illegal alcohol available for sale and that making *tari* is less laborious than collecting fresh date palm sap because harvesters do not need to clean and dry the earthen pots after each collection. They added that making *tari* is more profitable than selling fresh date palm sap. During our visit to communities near to the affected areas in Rangpur, the price of fresh date palm sap was $0.20 per liter, and the price of *tari* was $0.50 per liter. The price differential provides an incentive for making *tari*.

We observed bat roosts in the affected community of Rangpur, and date palm sap harvesters reported that they frequently observe bats flying near the date palm trees. The harvesters reported that they often find bat excreta in and on the sap pots. The harvesters reported filtering *tari* with a net or cloth before selling it to remove the excreta. In Rajshahi, the villagers reported that there is no bat roost in their community. However, they reported seeing bats visiting date palm trees at night. None of the harvesters in Rajshahi reported filtering *tari* before selling it to consumers.

## Discussion

The laboratory, clinical, and qualitative findings in this study suggest that the 14 case-patients in the 3 clusters we investigated were infected with NiV. The primary NiV case-patients identified in the clusters drank *tari* regularly in the evenings before their illness onsets, and none of them had a history of fresh date palm sap consumption or any exposure to other NiV case-patients, which were the main transmission pathways for NiV infection identified in previous outbreak investigations in Bangladesh (*9*,*26*). Moreover, none of the case-patients had exposure to sick animals, another possible pathway for NiV transmission reported in studies conducted in Malaysia and Singapore (*27*).

*Tari* is a date palm sap product. Because *tari* fermentation was a continuous process and date palm sap was fermented inside the *tari *pots while they were hanging in the trees, some date palm sap added to the *tari* might technically be fresh sap. However, the primary case-patients probably did not consume fresh date palm sap because *tari* was collected from 8 a.m. to noon but the primary case-patients drank *tari* only in the evening, which suggests that all the sap they consumed was at least partially fermented by the time of consumption. Findings from this investigation suggest that drinking *tari* is a potential source of NiV infection in Bangladesh. Investigators in India had similar findings during an outbreak reported in 2007 in West Bengal near the border with Bangladesh (*4*). They reported that drinking fermented date palm sap possibly contaminated with bats excreta and secretions was the source of NiV infection for the index patient, which further supports our assertion that NiV infection from drinking *tari* is plausible (*4*). Moreover, 2 clusters in this study were traced back to the same *tari* production village in Rangpur, further strengthening the conclusion that *tari* was the mode of transmission.

Previous studies have shown that fruit bats frequently lick the date palm sap and occasionally urinate inside collection pots (*14*). In our investigation, the reported evidence of bats visiting date palm trees, the presence of bat excreta inside *tari* pots, the reported use of the same pot for several days without cleaning, and the accumulation of sap from multiple pots into 1 pot suggest that sap is probably contaminated with bat urine or saliva during collection and fermentation. NiV can survive up to 4 days in bat urine and at least 1 day in sap contaminated with bat urine when kept at an average temperature of 19°C (*28*). A study to determine viability of NiV in artificial palm sap contaminated with NiV (strain Bangladesh/200401066) found no statistically significant reduction in NiV titers for at least 7 days when kept at a temperature of 22°C (*29*). Generally, enveloped and thus lipophilic viruses like NiV are susceptible to alcohol. A 60%–70% alcohol solution is recommended for sterilizing contaminated objects (*30*). A study conducted in India showed that *tari* derived naturally from fermenting date palm sap contains 5%–8% alcohol and has a pH of 4.5–6.0 (*12*). This 5%–8% alcohol concentration might not have been high enough or sufficiently distributed throughout the *tari* to sterilize the NiV, thus allowing persistence of viable virus and transmission of NiV to *tari* consumers during the winter months, when the ambient temperature ranges from 15°C to 28°C (*21*).

In Bangladesh, family caregivers commonly provide close-contact care to hospitalized patients (*31*). Infected patients often shed the virus through body secretions and excretions and can contaminate foods and surfaces, including bed rails, bed sheets, and towels (*32*,*33*). Close contact is the most likely route through which family caregivers became infected (*26*). Family caregivers identified in this study had direct contact with primary case-patients and their body secretions.

The findings of our study are subject to limitations. First, some participants may have been reluctant to report production, consumption, and selling of *tari* because these are illegal activities in Bangladesh. Initially, some of the date palm sap harvesters and *tari* producers were reluctant to share information with us. However, the social scientists on the investigation team built rapport and trust with the respondents, which encouraged the respondents to share sensitive information (*23*). Thus, the hesitance to disclose behaviors related to *tari* probably did not have a substantial effect on our findings. Second, no control group was available. Without a comparison group, we were unable to determine if our primary case-patients were more likely than other persons residing in these villages to drink *tari*. However, given the absence of evidence that the primary case-patients had other contact with bats, sick animals, or persons with NiV infection, consumption of *tari* appears to be the most likely transmission route. Third, we interviewed family members and friends of the deceased case-patients as proxy respondents to ascertain case exposures. However, during outbreaks of fatal diseases such as NiV infection (with a case-fatality rate >70%), there is no alternative to this approach (*20*). Since 2003, we have interviewed proxy respondents for case-exposures in every NiV-associated outbreak investigation conducted in Bangladesh (*3*,*9*,*19*). Fourth, because of the delays in investigation of the 2011 cluster, our definition for a confirmed cases of NiV infection was based on the presence of NiV IgM or IgG in serum samples. Confirming a case based on the presence of IgG is reasonable, however, because all the family caregivers from the 2011 Rangpur cluster had illness onset within 2 weeks after contact with a case-patient (*19*).

Because harvesting date palm sap for *tari* production is similar to harvesting it for consumption of fresh date palm sap, the intervention of using bamboo skirts to cover the shaved part of the date palm tree and the sap collection pots to prevent bat contact and possible NiV introduction is worth exploring. The use of bamboo skirts is already a successful, affordable, and culturally acceptable method to prevent bat access to date palm sap, and this strategy could also be used to prevent NiV transmission from *tari* consumption (*34*,*35*). In addition, *tari* harvesters from ethnic minority communities have limited access to mass media because of their ethnic, religious, and linguistic minority status in Bangladesh. Efforts should be made to raise their awareness about strategies that interrupt bat access to date palm sap. At this time, we are not aware of any studies that have tested the survival of NiV in *tari*. As a next step, we recommend testing NiV survival in *tari* at different levels of alcohol concentration.

All 3 of the clusters of NiV infection that we investigated were linked to drinking *tari*. Drinking *tari* might also be a route of exposure for other batborne viruses. A total of 55 newly described viruses from 7 virus families were recently identified in urine and saliva from *Pteropus* spp. bats in Bangladesh (*36*), suggesting that these bats could also contaminate *tari* with other viruses that could cause disease in humans. Date palm sap is harvested for fermentation in many areas where *Pteropus* spp. bats and other fruit bats are native, including Australia, Asia, and Africa (*37*–*40*). Consumers of fermented drinks and other date palm products that are harvested using similar processes as in Bangladesh might be at risk for NiV infection and other batborne diseases (*12*,*13*,*36*).
